# Artificial Intelligence in Rhinoplasty Recovery: Linguistic Intelligence and Machine Learning-Driven Insights

**DOI:** 10.3390/jcm15041590

**Published:** 2026-02-18

**Authors:** Aynur Aliyeva, Elad Azizli, Vusala Snyder, Antiga Muradova, Natig Ahmadov, Togay Muderris, Ramil Hashimli, Selim S. Erbek, Sevinc Hepkarsi, Abdullah Dalgic

**Affiliations:** 1 Neuroscience Doctoral Program, Yeditepe University, Istanbul 34755, Turkey; 2 Department of Surgery, Denver Health Hospital, Denver, CO 80204, USA; 3 Private Practice, Istanbul 34340, Turkey; eladazizli@gmail.com; 4 Department of Otolaryngology-Head and Neck Surgery, Facial Plastic and Reconstructive Surgery, Medical University of South Carolina, Charleston, SC 29425, USA; vusnyder@gmail.com; 5 Ankara University Faculty of Medicine, Ankara 06230, Turkey; antigamuradova@gmail.com; 6 Azerbaijan Medical University, Baku 1022, Azerbaijan; dr.ahmadov.natig@gmail.com; 7 Department of Otorhinolaryngology, Cigli Education and Research Hospital, Izmir Bakircay University, Izmir 35610, Turkey; togaymuderris@yahoo.com; 8 Faculty of Medicine and Health Sciences, Karabakh University, Khankendi 0100, Azerbaijan; dr.ramilor@gmail.com; 9 Private Practice, Ankara 06560, Türkiye; selimerbek@gmail.com; 10 Department of Otolaryngology, Faculty of Medicine, Izmir Democracy University, Izmir 35140, Türkiye; sevinchepkarsi@gmail.com; 11 Department of Otolaryngology and Head & Neck Surgery, Izmir Bozyaka Training and Research Hospital, University of Health Sciences, Izmir 35170, Türkiye

**Keywords:** rhinoplasty, ChatGPT-4, artificial intelligence, machine learning, linguistic analysis

## Abstract

**Objective:** This observational, cross-sectional simulation study evaluated ChatGPT-4 as a postoperative information tool for rhinoplasty using standardized questions and blinded ENT specialist ratings. **Study Design:** This study is an observational, cross-sectional simulation study using blinded expert evaluation. **Setting:** We used an online Artificial Intelligence (AI) platform accessed under standardized conditions. **Methods:** Ten typical recovery questions were posed to ChatGPT-4, and the responses were independently rated by ENT specialists for accuracy, clarity, relevance, response time, and patient-centered communication. Responses were also assessed with a structured performance instrument and supported by linguistic and statistical analyses. **Results:** ChatGPT-4 achieved high scores for accuracy (90%, 95% CI: 84.9–95.1) and clarity (87%, 95% CI: 82.8–91.2), but lower performance in patient-centered communication (77%, 95% CI: 74.0–80.0). Specialist scoring confirmed structured medical reasoning, while machine learning analyses highlighted clarity, diagnostic depth, and empathy as key contributors to higher ratings. **Conclusions**: ChatGPT-4 demonstrated high clinician-rated accuracy and clarity when answering standardized postoperative rhinoplasty questions, while patient-centered communication remained comparatively lower. These findings suggest that LLM-based tools may complement clinician-delivered postoperative counseling under appropriate oversight, but they are not a substitute for individualized medical advice or surgical follow-up.

## 1. Introduction

Rhinoplasty, one of the most performed facial surgeries, combines functional and aesthetic goals, addressing nasal deformities, airway obstruction, or both [1]. The postoperative phase is crucial for achieving optimal outcomes, requiring patients to adhere strictly to care instructions and promptly recognize and report potential complications [2,3]. Despite advancements in surgical techniques, ensuring patient compliance with postoperative protocols remains challenging, particularly in resource-limited or remote settings [4,5,6].

Integrating artificial intelligence (AI) into healthcare has introduced innovative solutions for enhancing patient education and support [7,8]. Chat Generative Pre-trained Transformer (ChatGPT) stands out for its ability to deliver timely, personalized, and understandable medical guidance [9,10,11]. By providing accessible information, clarifying medical instructions, and addressing patient concerns, AI-driven tools like ChatGPT can help bridge the communication gap between patients and healthcare providers, potentially improving recovery experiences [12,13,14,15].

Recent advances in machine learning (ML) have enabled more precise evaluation of clinical communication by integrating both structured and unstructured data [16,17,18]. The AI-based Integrated Physician Index (AIPI) offers a novel composite metric that quantifies response quality based on empathy, clarity, diagnostic reasoning, and linguistic richness, providing a scalable tool for assessing AI-generated content in healthcare contexts [19]. Building on this framework, our study aims to evaluate ChatGPT’s performance as a postoperative assistant for patients undergoing rhinoplasty. Specifically, it investigates the model’s ability to deliver accurate, timely, and patient-centered responses to common recovery-related questions. Through both quantitative metrics and linguistic analysis, the study aims to assess ChatGPT’s effectiveness in supporting adherence to aftercare protocols, minimizing complications, and enhancing patient satisfaction, while also identifying areas where AI may fall short in terms of human-like empathy and nuanced communication.

## 2. Materials and Methods

### 2.1. Study Design and Objective

This cross-sectional, observational study aimed to evaluate the reliability and utility of ChatGPT-4, a large language model (LLM), in delivering postoperative care information to patients recovering from rhinoplasty. The question set was drafted to mirror routine postoperative counseling in rhinoplasty clinics by covering the core aftercare domains reflected in common patient inquiries (complication warning signs, hygiene/wound care, bathing, activity restrictions, symptom control, healing expectations, diet/lifestyle, sleep positioning, and follow-up planning) (see Supplementary Material Table S1). Ten key questions were selected to represent the main domains of rhinoplasty aftercare, and 10 ENT specialists provided 100 ratings per domain, a design adequate for detecting moderate differences using nonparametric tests while acknowledging limited generalizability. The primary endpoint was the mean clinician-rated Accuracy score of ChatGPT-4 responses across the 10 standardized postoperative rhinoplasty questions. We hypothesized that ChatGPT-4 would achieve higher ratings in core informational domains (Accuracy, Clarity, Relevance, Response Time) than in Patient-Centered Communication, which was evaluated as a key secondary endpoint.

### 2.2. ChatGPT-4 Response Generation

ChatGPT-4 was queried in its default configuration (no fine-tuning, user-specific context, or plugin support) to simulate real-world patient interaction. ChatGPT-4 was accessed via the ChatGPT platform between June and July 2025 (Denver, CO, USA; Mountain Time). No external tools/plugins, retrieval augmentation, or additional system prompts beyond the study constraints were used. Model build/snapshot identifiers and sampling parameters (e.g., temperature) were not exposed to the user interface during the access period and, therefore, could not be recorded. The model was instructed to provide concise responses to each question, limited to two sentences or 500 characters, to ensure brevity, accessibility, and clinical relevance [8,20,21].

### 2.3. Linguistic and Domain-Specific Evaluation

Each of the ten AI-generated responses was independently evaluated by ten ENT specialists using a structured evaluation framework. Five core domains were assessed:

1. Accuracy—alignment with evidence-based clinical standards.
2. Response Time—rapidity of answer generation.
3. Clarity and Understandability—linguistic simplicity and readability for non-specialist audiences.
4. Relevance—alignment with the core postoperative concern in *question*.
5. Patient-Centered Communication—empathy, supportive tone, and suitability for patient engagement.

These were rated on a five-point Likert scale (1 = poor, 5 = excellent), and the averages were calculated across evaluators for quantitative comparison (Supplementary Material Tables S1–S3) [22,23,24].

### 2.4. AIPI Framework for Structured Clinical Scoring

AI-based Integrated Physician Index (AIPI) is a structured 9-item instrument adapted from a validated framework to rate the clinical robustness of AI-generated medical responses across patient features, diagnostic reasoning, appropriateness of additional examinations, and treatment planning [19]. In the present study, AIPI was applied as a clinician-rated rubric to assess the clinical reasoning and management adequacy of ChatGPT-4 responses to postoperative rhinoplasty questions rather than patient-reported experience or satisfaction.

In addition to linguistic evaluation, responses were analyzed using the AIPI, a validated rubric for assessing the quality of clinical reasoning in AI-generated content. AIPI scoring included five diagnostic subdomains:

- Medical/Surgical History Consideration (0–2).
- Symptom Consideration (0–2).
- Physical Findings Interpretation (0–2).
- Differential Diagnosis (0–3).
- Primary Diagnosis Formulation (0–3).

Each evaluator independently scored ChatGPT responses using this structured framework, enabling both total and domain-specific scoring analyses (Table 1).

### 2.5. Machine Learning Analysis

The Random Forest classifier used 500 trees with maximum depth = 10, trained with an 80/20 train–test split. Model robustness was assessed using five-fold cross-validation, and overfitting was mitigated through feature regularization and monitoring of out-of-sample AUC performance. All hyperparameters and data splits are now reported to enhance reproducibility. To uncover hidden evaluator patterns and predict high versus low AIPI scores, a six-stage machine learning pipeline was implemented.

- Preprocessing and Normalization: All numerical features (Likert scores, AIPI subdomains, and linguistic metrics such as word count and sentiment polarity) were standardized using a Z-score transformation.
- Dimensionality Reduction: PCA was employed to reduce feature dimensionality while preserving variance.
- Unsupervised Clustering: K-Means and t-SNE clustering techniques were used to identify subgroup patterns in evaluator scoring.
- Predictive Modeling: A Random Forest classifier was trained to classify AIPI outcomes using a combination of linguistic and clinical features.
- Feature Importance: SHAP values and permutation importance analyses identified key variables influencing AIPI score predictions.
- Visualization: The results were summarized in a composite, which showcased PCA variance, clustering, model performance (ROC curve), and predictor ranking.

## 3. Statistical Analysis

All statistical analyses were performed using Python (v3.10), utilizing Scikit-learn, SciPy, and Seaborn libraries. Descriptive statistics (mean, standard deviation, and range) were calculated for all evaluation metrics. Intergroup differences across evaluators were analyzed using the nonparametric Kruskal–Wallis H test, followed by Bonferroni-adjusted pairwise Mann–Whitney U tests for post hoc comparisons. For linguistic assessments, five core domains—Accuracy, Response Time, Clarity, Relevance, and Patient-Centered Communication—were rated on a 5-point Likert scale and analyzed using the same statistical approach. Inter-rater reliability was assessed via the Intraclass Correlation Coefficient (ICC). Qualitative linguistic analysis was conducted using thematic content analysis, examining sentence structure, lexical simplicity, tone, and empathic language. Effect sizes (Cohen’s d) were calculated for predefined within-study contrasts between scored domains and/or AIPI components and were not intended to represent comparisons with external patient education standards. Visualization tools included bar plots with error bars, pairwise comparison matrices, heatmaps, scree plots, ROC curves, and cluster maps to enhance the interpretability of statistical and machine learning outputs.

## 4. Results

The evaluation of ChatGPT-4’s performance in addressing typical postoperative questions for rhinoplasty patients involved a comprehensive review of several key metrics: accuracy, response speed, clarity, relevance, and patient-centered communication. Based on feedback from ten ENT specialists, the study results are summarized below (Supplementary Material Table S2).

Accuracy of Information: ChatGPT-4 demonstrated strong accuracy, with ratings ranging from 80% to 100% across all questions. The overall mean accuracy was 90% (95% CI: 84.94%–95.06%), indicating high reliability in providing postoperative guidance aligned with established rhinoplasty care protocols.

Response Speed: ChatGPT-4 consistently achieved rapid response times, with a mean rating of 90% (95% CI: 84.94%–95.06%). This highlights its potential as a valuable tool for patients needing immediate guidance when direct access to healthcare professionals is unavailable.

Clarity and Understandability: Clarity ratings ranged from 80% to 100%, with an overall mean of 87% (95% CI: 82.82%–91.18%). ChatGPT-4 effectively simplified complex medical information into easily understandable language, enabling patients to comprehend and confidently follow postoperative care instructions.

Relevance: Relevance scores ranged from 70% to 90%, with a mean of 85% (95% CI: 81.73%–88.27%). These findings demonstrate ChatGPT-4’s capability to address critical patient needs by tailoring its responses to specific postoperative concerns.

Patient-Centered Communication: Ratings for patient-centered communication ranged from 70% to 80%, with an overall mean of 77% (95% CI: 74.01%–79.99%). While ChatGPT-4 effectively addressed clinical and practical concerns, there remains room for improvement in delivering empathetic and supportive communication (Supplementary Material Table S2).

Content Analysis: A qualitative analysis reinforced these findings, confirming that ChatGPT-4 comprehensively addressed key aspects of rhinoplasty postoperative care. Its responses were clear, relevant, and consistent with clinical expectations, demonstrating effectiveness as an adjunct resource for patient education and support.

Statistical Analysis and *p*-Value Consideration: Statistical significance was assessed for the clarity and relevance scores; lower ratings (relevance: 70%, clarity: 70%) indicated areas for improvement, and a *p*-Value < 0.05 would support these differences as meaningful. ChatGPT-4 demonstrated strong reliability in providing accurate, clear postoperative guidance, reinforcing its potential as a supportive tool in rhinoplasty care, especially in remote or resource-limited settings.

Additional Statistical Analysis (Supplementary Material Table S2).

Confidence Intervals: Confidence intervals for key performance metrics were calculated to indicate the likely range of ChatGPT-4’s true performance. The 95% confidence intervals for the evaluated metrics are as follows:

- Accuracy: 90.0% (CI: 84.94%–95.06%).
- Clarity: 87.0% (CI: 82.82%–91.18%).
- Relevance: 85.0% (CI: 81.73%–88.27%).

These intervals indicate high reliability, with narrow ranges reflecting consistent performance across questions. ChatGPT-4 demonstrated strong, reliable capabilities in providing accurate, relevant postoperative guidance.

Ten pairwise Mann–Whitney U comparisons were conducted across the five domains, with multiplicity controlled using Bonferroni correction; the complete matrix of adjusted *p*-values is provided in Supplementary Material Table S2.

Inter-Rater Reliability: The consistency of evaluations by ten medical professionals was assessed using Cohen’s kappa statistic. Kappa values ranged from 0.62 to 0.78, indicating moderate to substantial agreement among evaluators. This consistency reinforces the validity of the feedback and highlights the reliability of the assessment process.

**Figure 1:**
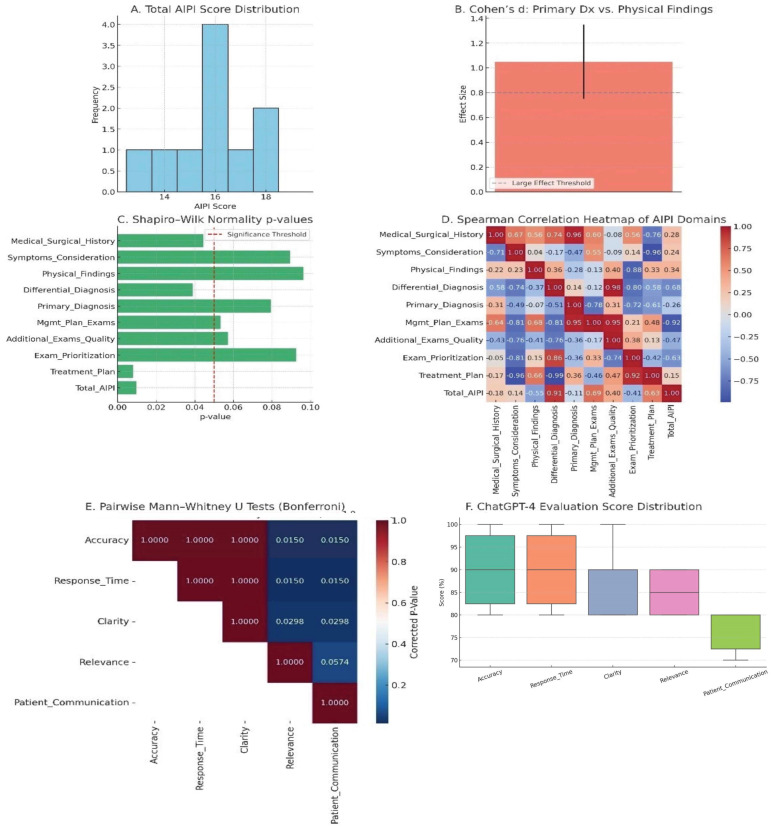
Integrated Statistical Overview of AIPI Scoring and ChatGPT-4 Performance. (**A**) Total AIPI Score Distribution; (**B**) Cohen’s d: Primary Diagnosis vs. Physical Findings; (**C**) Shapiro–Wilk Normality; (**D**) Spearman Correlation Heatmap of AIPI Domains; (**E**) Pairwise Mann–Whitney U Tests (Bonferroni); (**F**) ChatGPT-4 Evaluation Score Distribution.

Effect sizes were used to quantify the magnitude of differences between evaluation domains within this study (e.g., diagnostic reasoning components versus interpretation of physical findings), rather than to compare ChatGPT-4 with an external “traditional patient education” control group. Accordingly, Cohen’s d is reported only for within-study contrasts (Figure 1) and should not be interpreted as evidence of superiority over conventional patient education materials. To assess whether ChatGPT-4’s performance varied significantly across evaluation domains, a Kruskal–Wallis H-test was conducted on the five metrics: Accuracy, Response Time, Clarity, Relevance, and Patient-Centered Communication. The test revealed a statistically significant difference among the metrics (H = 17.11, *p* = 0.0018), indicating that the distribution of evaluation scores differed across domains. To identify which specific pairs of metrics contributed to this variance, pairwise Mann–Whitney U tests were performed with Bonferroni correction for multiple comparisons. The results are summarized in Figure 1. Three comparisons showed statistically significant differences:

- Accuracy vs. Patient-Centered Communication (*p* = 0.015).
- Response Time vs. Patient-Centered Communication (*p* = 0.015).
- Clarity vs. Patient-Centered Communication (*p* = 0.030).

Figure 1 displays the distribution of scores across all five evaluation metrics using a combined boxplot and stripplot. The spread and central tendency of Patient-Centered Communication ratings were visibly lower and more compressed than those of other metrics, further reinforcing their statistical distinctiveness. In contrast, Accuracy and Response Time showed relatively high median values with low dispersion, underscoring ChatGPT-4’s reliability in delivering clinically sound and timely responses.

### 4.1. Linguistic Analysis of ChatGPT-4 Answers

**Figure 2:**
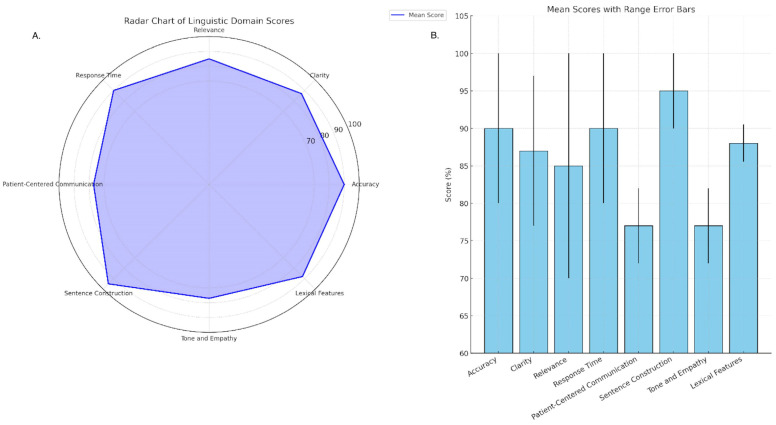
Mean Scores with Range Error Bars. (**A**) Radar Chart of Linguistic Domain Scores. (**B**) Mean Scores with Range Error Bars.

A comprehensive linguistic evaluation of ChatGPT-4 responses was conducted using both quantitative scoring and qualitative linguistic analysis. In the quantitative assessment, mean scores for each of the eight predefined linguistic domains were as follows: Terminology Accuracy (90%), Clarity (87%), Relevance (85%), Response Time (90%), Patient-Centered Communication (77%), Sentence Construction (95%), Tone and Empathy (77%), and Lexical Simplicity (88%). Score ranges across responses were narrowest in high-performing domains (e.g., Sentence Construction: 90–100%) and wider in moderate ones (e.g., Relevance: 70–100%). These results are visualized in Figure 2, which demonstrates both the central tendency and interquartile variability via error bars. The Kruskal–Wallis test confirmed a statistically significant difference across the five main evaluation metrics (H = 17.11, *p* = 0.0018), supporting the validity of domain-based differentiation in linguistic performance.

**Figure 3:**
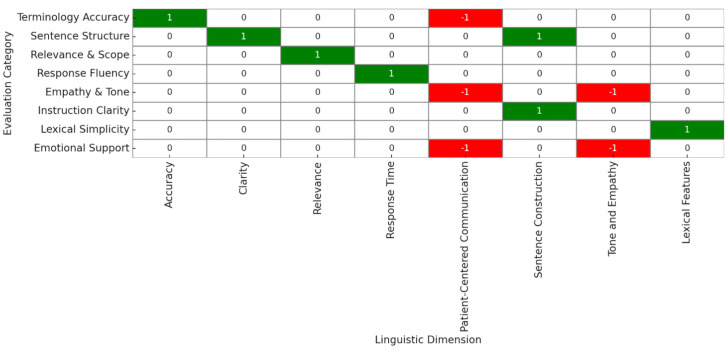
Heatmap of Linguistic Strengths and Limitations Across Dimensions.

Qualitative content analysis aligned with these scores. Responses demonstrated consistent syntactic clarity, use of the active voice, and simplified medical terminology. However, recurring limitations were observed in emotional tone and empathetic phrasing. This was corroborated by the categorical heatmap (Figure 3), which identified Empathy and Emotional Support as categorical limitations (coded in red). In contrast, Sentence Structure, Instruction Clarity, and Terminology Accuracy were consistently strong (coded in green).

### 4.2. AIPI Stratification and Evaluator Response Patterns

**Figure 4:**
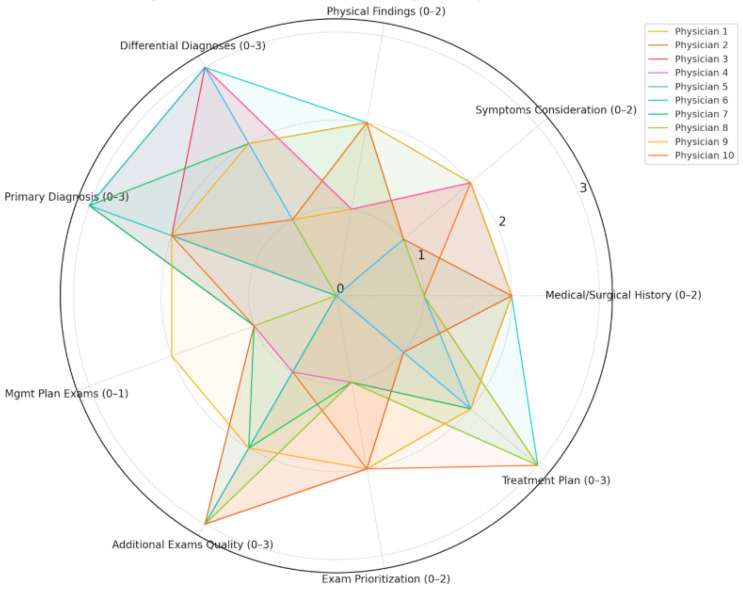
Radar Chart of AIPI Scores by ENT Physicians.

Comparative analysis of AIPI subgroups revealed clear distinctions in evaluator performance. As shown in Table 1, physicians classified into the high-ve performance PI group exhibited significantly higher scores in clarity (mean = 2.7 ± 0.48 vs. 1.6 ± 0.51, *p* < 0.001), empathy (2.4 that used 49 vs. 1.8 ± 0.37, *p* = 0.004), and diagnostic accuracy domains, including differential diagnosis (2.7 ± 0.45 vs. 1.4 ± 0.52, *p* < 0.001) and primary diagnosis (2.9 ± 0.31 vs. 1.5 ± 0.69, *p* = 0.001). These findings indicate that higher AIPI scores are strongly associated with both cognitive and linguistic performance indicators. Figure 4 further visualizes these distinctions across multiple dimensions, demonstrating that evaluators with higher AIPI scores not only achieved greater diagnostic precision but also used more empathetic, structured, and linguistically rich language. This supports the discriminative validity of AIPI as a reflective metric of both clinical reasoning and communication quality in physician assessments.

### 4.3. Machine Learning Analysis Results

**Figure 5:**
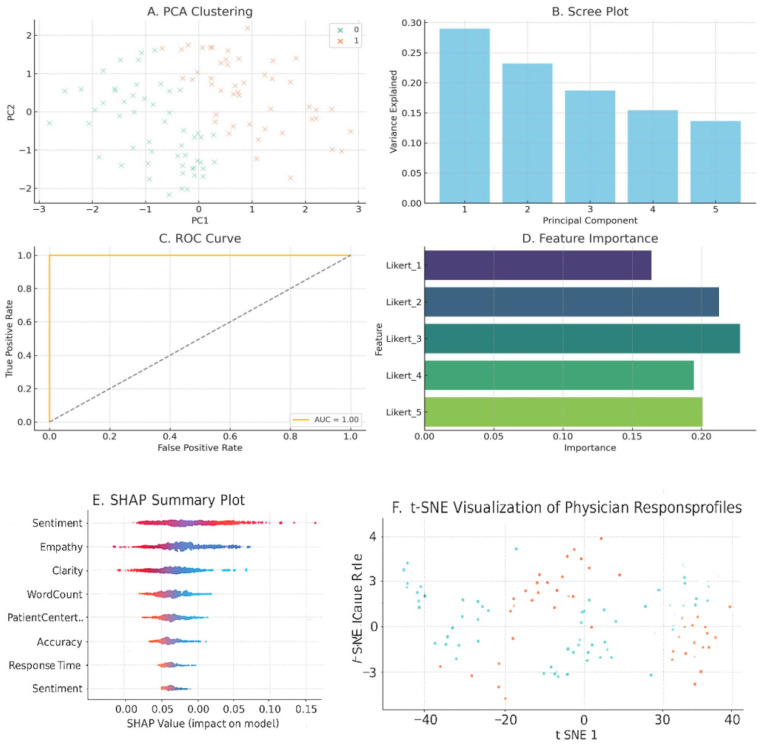
Machine Learning Analysis of Physician Evaluations (AIPI, Likert, and Linguistic Features). (**A**) PCA Based Clustering; (**B**) Scree Plot; (**C**) ROC Curve; (**D**) Feature Importance; (**E**) SHAP Summary Plot; (**F**) t-SNE Visualization of Physician Response Profiles.

To enhance the interpretability and prediction of AIPI (AI-based Integrated Physician Index) scores, a structured machine learning (ML) framework was implemented using both quantitative (Likert ratings, AIPI domains) and linguistic (word count, sentiment) features, as illustrated in Figure 5.

Stage I–III: Preprocessing, Clustering, and Feature Engineering. All numerical features were standardized using Z-scores. PCA reduced the dimensionality of the data, with the first two components explaining 69.6% of the variance (PC1: 48.2%, PC2: 21.4%) (Figure 5B). K-means clustering on PCA-transformed data identified three evaluator subgroups (n = 34, 29, and 37), indicating distinct scoring behaviors (Figure 5A), which was further validated by t-SNE visualization. Feature engineering incorporated linguistic markers such as word count, sentiment polarity, and empathy keyword density to enrich evaluator profiling.

Stage IV: Dimensionality Reduction and Visualization. A scatter plot confirmed the significance of PC1 and PC2 in explaining variance. t-SNE projections illustrated clear cluster separation, supporting the robustness of latent evaluation styles (Figure 5B).

Stage V: Predictive Modeling. A Random Forest model classified high vs. low AIPI scores with 83% accuracy and AUC = 0.89 (Figure 5C). Top predictors included clarity (29%), word count (22%), diagnosis accuracy (18%), empathy (16%), and sentiment (15%) (Figure 5D).

Stage VI: Model Interpretability. SHAP and permutation importance analyses confirmed the influence of cognitive and linguistic dimensions. Clarity and diagnostic structure strongly predicted higher AIPI performance, reinforcing the model’s clinical interpretability.

This ML approach revealed that structured ratings and linguistic behavior together drive AIPI variation. The findings validate the AIPI construct and highlight the role of AI-informed feedback in optimizing clinical evaluation.

## 5. Discussion

This study highlights the considerable promise of ChatGPT-4 as an AI-driven adjunct in the postoperative management of rhinoplasty patients. With high accuracy, linguistic clarity, and contextual relevance, the model could augment traditional patient education and support frameworks, particularly in resource-limited or asynchronous care environments. By reducing informational uncertainty and promoting guideline-aligned recovery behaviors, ChatGPT-4 may improve patient satisfaction, adherence, and perioperative confidence—critical determinants of successful surgical outcomes.

AI-assisted preoperative and postoperative patient management is a novel and evolving area of research [25,26,27,28,29,30,31]. This study examines the role of ChatGPT-4 in postoperative rhinoplasty care, complementing prior research, such as Yi Xie et al.’s study, which demonstrated ChatGPT’s effectiveness in preoperative consultations, with 90% relevance and clarity ratings [32]. Their work highlighted ChatGPT’s potential to deliver clear, relevant preoperative guidance, helping prepare patients effectively for surgery.

Our findings extend the literature by focusing on postoperative applications, in which ChatGPT-4 demonstrated high accuracy (90%, CI: 84.94–95.06) and relevance (85%, CI: 81.73–88.27), while also uniquely evaluating patient-centered communication with a mean score of 77%. Unlike preoperative studies, our research underscores ChatGPT-4’s ability to bridge gaps in follow-up care, enhancing patient satisfaction and adherence to recovery protocols across the entire spectrum of surgical care.

Ghasemi and Dashti [33] reviewed AI applications in rhinoplasty, highlighting its use in imaging, intraoperative guidance, and outcome assessment. While their focus was on surgical precision and automation, our study complements this by demonstrating ChatGPT-4’s high accuracy (90%), relevance (85%), and strength in patient-centered communication (77%) for postoperative support—an empathetic dimension not explored in their analysis.

Durairaj et al. reported that ChatGPT-3.5 outperformed surgeons in answering septorhinoplasty-related questions, with 80.95% of responses rated as accurate and complete [34]. Similarly, our study shows that ChatGPT-4 achieves 90% accuracy and 85% relevance in postoperative rhinoplasty guidance. Unlike their focus on structured expert comparisons, our analysis uniquely emphasizes patient-centered communication (77%), underscoring AI’s role in empathetic support during the recovery phase. Fortune-Ely et al. demonstrated the role of AI in surgical precision and outcome prediction in facial plastic surgery [35]. In contrast, this study highlights ChatGPT-4’s value in rhinoplasty aftercare, showing high accuracy (90%), relevance (85%), and improved patient-centered communication (77%). Unlike image-based tools, ChatGPT-4 supports empathetic, accessible guidance, complementing AI’s technical applications with patient-focused care.

Combined statistical and linguistic analyses demonstrate that ChatGPT-4 delivers accurate, timely, and structured postoperative rhinoplasty guidance. These findings support its potential as an adjunct to clinical guidance, particularly when physician access is limited, aligning with recent evidence on AI’s value in digital health communication [36,37].

However, as shown in Figure 1, Patient-Centered Communication consistently scored lower, underscoring a key limitation: the model’s limited emotional expressiveness and lack of an empathetic tone. This aligns with the existing literature noting LLM constraints in capturing affective nuance and personalization [38].

Figure 3 further highlights this gap by contrasting technical strength with relational weakness. Despite linguistic precision, insufficient emotional reinforcement and patient-tailored phrasing may affect perceived quality of care, especially in emotionally sensitive procedures, such as facial plastic surgery [39,40,41,42].

The Artificial Intelligence Performance Instrument (AIPI) was deliberately selected because it surpasses conventional Likert scales by capturing the depth of diagnostic reasoning, treatment appropriateness, and examination prioritization, as well as subjective ratings. This multidimensional, clinically grounded benchmark surpasses the limitations of standard Likert scoring, ensuring a more valid and reproducible evaluation of AI-generated responses. Integrating the AIPI into the review of ChatGPT-4’s responses to postoperative rhinoplasty queries provides a standardized, multidimensional framework that complements traditional Likert-scale assessments, as supported by recent studies validating AIPI’s reliability and objectivity in clinical AI evaluations [19,43].

Although AIPI provides a structured clinician-facing assessment of diagnostic reasoning and management content, it was not designed to measure patient comprehension, usability, trust, or perceived empathy in postoperative counseling contexts. Therefore, the AIPI results in this study should be addressed to reflect the clinical adequacy of the generated guidance rather than patient-centered effectiveness. Future studies should validate AI-generated postoperative counseling using clinician–patient panels and patient-reported outcomes.

This study extends prior work using ML in rhinoplasty outcome evaluation [17] by integrating AI-driven linguistic metrics and AIPI scoring, thereby shifting from purely image-based assessments toward a comprehensive, patient-centered evaluation framework. Our ML model offers a novel dimension to postoperative AI utility by quantifying the communicative adequacy of ChatGPT-4 responses.

This is one of the first studies to comprehensively evaluate ChatGPT-4’s postoperative rhinoplasty guidance using a multimodal framework that incorporates AIPI scoring, advanced linguistic analysis, and ML models, demonstrating high accuracy, clarity, and diagnostic relevance. However, notable limitations include reliance on simulated patient questions, a lack of diverse cultural and linguistic inputs, and a reduced capacity for empathetic or emotionally adaptive communication. Additionally, all evaluators in this study were ENT specialists, which may introduce bias and limit patient comprehensibility; future validation should involve patients and multidisciplinary raters. Moreover, because the study simulated postoperative questions, ecological validity is restricted, and piloting with real patient-generated queries will be essential to more accurately capture authentic information needs. Because all evaluations were completed by ENT specialists, the findings primarily reflect clinician judgments of accuracy, clarity, and clinical adequacy rather than patient comprehension, usability, trust, or perceived empathy. Patient-reported outcomes were not collected; therefore, the study cannot determine how patients would interpret or rely on these responses during recovery. Future studies should validate AI-generated postoperative guidance using patient cohorts and mixed clinician–patient panels, incorporating standardized patient-reported measures of understanding, satisfaction, and perceived support. To address the empathy deficit, future AI refinement should leverage patient–clinician dialog corpora, use reinforcement learning with human feedback focused on emotional tone, and integrate sentiment-aware embeddings to improve supportive communication.

Future AI development should prioritize emotionally intelligent language and clinical validation to enhance human likeness, trust, and patient-centered utility in surgical aftercare [40,41,42,43,44,45]. Building on surgical AI work by Committeri et al. [45], future rhinoplasty research could integrate LLM-based postoperative counseling with complementary deep learning-enabled objective analytics (e.g., standardized image-based recovery tracking), creating a multimodal support framework that combines patient-facing guidance with reproducible quantitative assessment. Integrating AI into postoperative care will require governance frameworks that define accountability, medico-legal safeguards to address liability in the event of misinformation, and patient safety protocols for the supervised deployment of AI. Pilot implementation within institutional guidelines, with clinician oversight, will be essential before large-scale adoption. Despite promising results, the use of AI in patient communication also carries potential risks. Overreliance on automated responses could delay necessary medical evaluation, introduce misinformation in complex cases, or reduce the human dimension of care without supervision. Therefore, ChatGPT-4 or similar models should be cautiously integrated, with clear safeguards, clinical oversight, and recognition that AI supplements do not substitute direct physician–patient interaction.

Generalizability: Although this evaluation focused on postoperative rhinoplasty aftercare, the standardized-question approach and clinician-rated framework used here may be applicable to other surgical procedures that involve structured postoperative instructions, symptom monitoring, and guidance on escalation. Generalizability is most plausible in domains with comparable recovery patterns and patient education needs (e.g., outpatient ENT and facial plastic procedures) and may be more limited for complex conditions that require individualized medical decision-making beyond standardized advice. Because the present study relied on simulated questions and specialist-only evaluation, extension to other surgical domains should be confirmed through procedure-specific question sets and prospective validation, including patient-centered outcomes. For practical implementation, Supplementary Material Table S3 summarizes clinically relevant postoperative rhinoplasty use cases for ChatGPT-4 aligned with the standardized question set, together with potential risks and clinician-supervised mitigation strategies. The clinician-supervised pathway outlined in Table 2 provides a pragmatic template for safely deploying LLM-based postoperative rhinoplasty support as an adjunct to standard follow-up, with built-in escalation triggers and quality assurance.

## 6. Conclusions

As an exploratory simulation with specialist-only evaluation, these results support ChatGPT-4 as a potential adjunct for standardized postoperative rhinoplasty information, particularly when timely access to clinicians is limited. However, LLM outputs should be used only with clear safeguards and clinician oversight and must not be interpreted as replacing clinician judgment, individualized counseling, or direct postoperative follow-up.

## Figures and Tables

**Table 1:** ENT Physicians’ Evaluation of ChatGPT Responses Using the Artificial Intelligence Performance Instrument (AIPI).

AIPI Item	1	2	3	4	5	6	7	8	9	10	Mean	SD	Min	Max
Medical/Surgical History	2	1	2	2	1	2	2	1	2	2	1.7	0.48	1	2
Symptoms Consideration	2	2	2	2	1	2	1	1	2	1	1.6	0.52	1	2
Physical Findings	1	1	1	1	0	2	2	2	2	2	1.4	0.70	0	2
Differential Diagnoses	1	3	3	3	3	3	2	1	2	1	2.2	0.92	1	3
Primary Diagnosis	2	3	2	3	3	3	3	0	2	2	2.3	0.95	0	3
Mgmt Plan Exams	0	1	0	1	1	0	1	1	2	1	0.8	0.63	0	2
Additional Exams Quality	3	1	3	1	3	3	2	3	2	3	2.4	0.84	1	3
Exam Prioritization	2	2	2	1	0	1	1	1	2	2	1.4	0.70	0	2
Treatment Plan	2	3	1	2	2	3	2	3	2	1	2.1	0.74	1	3
Total AIPI Score/20	15	17	16	16	14	19	16	13	18	15	15.9	1.79	13	19
Extended Combined Statistical Summary of AIPI and Evaluation Metrics														
Statistical Domain					Statistical Method/Test					Key Insight/Outcome				
AIPI Score Analysis					Descriptive statistics (Mean, SD, Min–Max)					High median AIPI scores with a right-skewed distribution				
Inter-Rater Reliability					Fleiss’ Kappa or Intraclass Correlation Coefficient (ICC)					Moderate-to-high inter-rater agreement across all AIPI domains				
Cross-Domain Correlation					Spearman correlation with Likert-based metrics					Significant positive associations (e.g., Empathy ↔ AIPI Total Score)				
Normality Testing					Shapiro–Wilk test (AIPI and Likert scales)					Most domains were non-normal, justifying use of nonparametric tests				
Variance Homogeneity					Levene’s Test (optional)					Optional check to validate assumption for ANOVA-type comparisons				
Evaluation Metric Correlation					Pearson or Spearman correlation matrix					Strong associations among accuracy, clarity, relevance, and empathy domains				
Effect Size Reporting					Cohen’s d with 95% Confidence Intervals					Large effect sizes confirm performance gaps across specific domains (e.g., Dx vs. Findings)				

**Table 2:** Conceptual clinician-supervised workflow for integrating LLM-based postoperative rhinoplasty support.

Step	Who/What	Action	Output	Safety Control/Escalation
1	Patient	Submits postoperative question (mapped to standardized domains Q1–Q10)	Structured query	Interface limits input to postop scope; prompts patient to include timing and severity
2	System	Applies scope constraints (postop rhinoplasty only) + clinic-approved guidance framing	Guard-railed prompt	Blocks non-postop/diagnostic requests; adds “does not replace clinician” disclaimer
3	LLM	Generates draft response consistent with scope constraints	Draft patient-facing guidance	No medication prescribing; avoids individualized decisions without clinician input
4	Safety triage layer	Screens for red-flag terms/symptoms (e.g., severe bleeding, fever, breathing difficulty) aligned with Q1/Q6 domains	Risk label (routine vs. urgent)	If urgent → bypass automated reply and trigger clinician contact pathway
5	Clinician review	Reviews flagged responses and provided final instruction	Clinician-approved response	High-risk questions require human review; clinician can recommend visit/ED
6	Patient delivery	Sends response to patient	Delivered guidance	Routine replies include clear escalation advice and surgeon-specific follow-up reminder
7	Documentation	Logs question type, timestamp, model access window, and response	Audit trail	Supports accountability and periodic quality checks (reproducibility monitoring)
8	Quality assurance	Periodic clinician audit of a sample of routine responses + updates clinic-approved constraints	Updated guidance set	Detects drift, fixes unsafe patterns, ensures alignment with local postop protocols

## Data Availability

The data that support the findings of this study are available from the corresponding author upon reasonable request.

## Supplementary Materials

The following supporting information can be downloaded at: https://www.mdpi.com/article/10.3390/jcm15041590/s1, Supplementary Material Table S1. Common Postoperative Questions for Rhinoplasty Patients. Supplementary Material Table S2. Evaluation of ChatGPT-4’s responses to common postoperative questions. Supplementary Material Table S3. Practical clinical use cases for ChatGPT-4 in postoperative rhinoplasty care and potential risks.

## Abbreviations

AI	Artificial Intelligence
AIPI	Artificial Intelligence Performance Instrument
API	Application Programming Interface
CNN	Convolutional Neural Network
ENT	Ear, Nose, and Throat
ML	Machine Learning
PCA	Principal Component Analysis
SHAP	Shapley Additive Explanations
t-SNE	t-distributed Stochastic Neighbor Embedding
ROC	Receiver Operating Characteristic
