# Supplementary material for: Artificial Intelligence in Rhinoplasty Recovery: Linguistic Intelligence and Machine Learning-Driven Insights
Source: J Clin Med. 2026 Feb 18;15(4):1590. doi: 10.3390/jcm15041590 (PMC12941429; doi:10.3390/jcm15041590)
Supplement: Supplementary file 1 [file jcm-15-01590-s001.zip › jcm-4126320-supplementary.pdf]

Supplementary Material Table S1: Common Postoperative Questions for Rhinoplasty Patients

Number	Questions
1	What signs of infection or complications should I monitor following rhinoplasty?
2	How should I care for my nose and surgical site, and what are the best hygiene practices?
3	When is it safe to wash my face or get my nose wet, and what precautions should I take while bathing?
4	What activities or environments should I avoid during my recovery?
5	How can I manage discomfort, swelling, or nasal congestion after surgery?
6	What symptoms would indicate that I need to contact my healthcare provider immediately?
7	How long will it take for my swelling to subside, and what can I expect during the healing process?
8	Are there any specific dietary recommendations or lifestyle changes that could aid in my recovery?
9	What precautions should I take while sleeping to ensure proper healing of my nose?
10	How often should I follow up with my healthcare provider, and what should I expect during these visits?

Supplementary Material Table S2: Evaluation of ChatGPT-4's responses to common postoperative questions.

Questions		ChatGPT-4's responses to common questions	Evaluation of ChatGPT's responses by ten medical professionals				
			Accuracy Of Information	Response Time	Clarity And Understandability	Relevance	Patient-Centered Communication
1	What signs of infection or complications should I monitor following rhinoplasty?	After rhinoplasty, monitor for signs of infection or complications such as persistent redness, swelling, or warmth around the nose, severe pain unrelieved by medication, fever over 100.4°F (38°C), unusual or foul-smelling discharge, excessive bleeding, or difficulty breathing. If you notice any of these symptoms, contact your healthcare provider immediately.	90% (9/10)	100% (10/10)	90% (9/10)	80% (8/10)	70 % (7/10)
2	How should I care for my nose and surgical site, and what are the best hygiene practices?	Keep your nose clean by gently dabbing with a clean, damp cloth if needed, avoiding any pressure. Use saline sprays as recommended to keep nasal passages moist. Do not remove crusts or touch the surgical site unnecessarily. Avoid blowing your nose, and follow your surgeon's instructions for cleaning and caring for the area.	80%(8/10)	90% (9/10)	100% (10/10)	90% (9/10)	80% (8/10)
3	When is it safe to wash my face or get my nose wet, and what precautions should I take while bathing?	You can usually wash your face gently 24-48 hours after surgery, avoiding the surgical site. Use a damp cloth to clean around the nose without applying pressure. Avoid submerging your nose in water or exposing it to hot steam during bathing. Follow your surgeon's specific timeline and instructions for care.	100% (10/10)	80% (8/10)	90% (9/10)	80% (8/10)	70 % (7/10)
4	What activities or environments should I avoid	Avoid strenuous activities, heavy lifting, bending over, or exercise for at least 2-4 weeks. Steer clear of smoky, dusty, or	80% (8/10)	90% (9/10)	90%(9/10)	80% (8/10)	80% (8/10)

	during my recovery?	polluted environments that could irritate your nose. Protect your nose from direct sunlight and avoid wearing glasses that rest on the nasal bridge until cleared by your surgeon.					
5	How can I manage discomfort, swelling, or nasal congestion after surgery?	Manage discomfort with prescribed pain relievers and cold compresses applied gently around the nose to reduce swelling. Keep your head elevated, even while sleeping, to minimize swelling. Use saline nasal sprays as recommended to relieve congestion, and avoid blowing your nose. Follow your surgeon's instructions closely for safe recovery	90% (9/10)	90% (9/10)	80% (8/10)	90% (9/10)	80% (8/10)
6	What symptoms would indicate that I need to contact my healthcare provider immediately?	Contact your healthcare provider immediately if you experience persistent or severe pain unrelieved by medication, excessive or prolonged bleeding, fever over 100.4°F (38°C), foul-smelling discharge, significant redness or warmth around the surgical site, difficulty breathing, or sudden changes in nasal shape. These could indicate complications requiring prompt attention.	80% (8/10)	90% (9/10)	90% (9/10)	80% (8/10)	80% (8/10)
7	How long will it take for my swelling to subside, and what can I expect during the healing process?	Most swelling subsides within 2-4 weeks, but subtle swelling can persist for up to a year. During healing, expect initial bruising, tightness, and nasal congestion, which improve over time. Full results become visible as swelling resolves, so patience is essential. Follow your surgeon's guidelines for the best recovery outcomes.	100% (10/10)	80% (8/10)	90% (9/10)	80% (8/10)	70 % (7/10)
8	Are there any specific dietary recommendations or lifestyle changes that could aid in my recovery?	Maintain a balanced diet rich in protein, vitamins C and A, and zinc to support healing. Stay hydrated, avoid salty foods to reduce swelling, and refrain from alcohol and smoking as they can delay recovery. Gentle, regular meals and good nutrition are key to promoting optimal healing. Follow your surgeon's	90%(9/10)	100% (10/10)	80% (8/10)	90% (9/10)	80% (8/10)

		advice for additional recommendations.					
9	What precautions should I take while sleeping to ensure proper healing of my nose?	Sleep on your back with your head elevated using extra pillows or a wedge to reduce swelling and prevent pressure on your nose. Avoid sleeping on your side or stomach to protect the surgical site. Use a neck pillow for added stability and follow your surgeon's advice for maintaining proper positioning.	100% (10/10)	80% (8/10)	80% (8/10)	90% (9/10)	80% (8/10)
10	How often should I follow up with my healthcare provider, and what should I expect during these visits?	Follow-up visits typically occur within the first week, then at 1 month, 3 months, and 6 months post-surgery, depending on your surgeon's plan. During these visits, your surgeon will monitor healing, remove sutures or splints if needed, address concerns, and provide guidance for the next recovery phase. Always adhere to the schedule advised.	90% (9/10)	100% (10/10)	80% (8/10)	90% (9/10)	80% (8/10)

Advance Statistical analysis of ChatGPT-4's responses Evaluation

Metric	Mean (%)	Standard Deviation (%)	95% CI Lower (%)	95% CI Upper (%)	IQR (%) (Q1-Q3)
Accuracy of Information	90.0	8.16	84.94	95.06	80.0-100.0
Response Time	90.0	8.16	84.94	95.06	80.0-100.0
Clarity and Understandability	87.0	6.75	82.82	91.18	80.0-90.0
Relevance	85.0	5.27	81.73	88.27	80.0-90.0
Patient-Centered Communication	80.0	4.83	74.01	80.0	70.0–80.0

CI = Confidence Interval; SD = Standard Deviation; All values reflect aggregated scores across ten evaluators.

Pairwise Mann–Whitney U tests across evaluation domains with Bonferroni correction (10 comparisons)

Pairwise comparison	Bonferroni-adjusted p-value

Accuracy vs Response Time	1.0000
Accuracy vs Clarity	1.0000
Accuracy vs Relevance	0.0150
Accuracy vs Patient-Centered Communication	0.0150
Response Time vs Clarity	1.0000
Response Time vs Relevance	0.0150
Response Time vs Patient-Centered Communication	0.0150
Clarity vs Relevance	0.0298
Clarity vs Patient-Centered Communication	0.0298
Relevance vs Patient-Centered Communication	0.0574

Supplementary Material Table S3. Practical clinical use cases for ChatGPT-4 in postoperative rhinoplasty care and potential risks

Use case domain	Study question (Q#)	Practical use in postop care	Potential risk	Mitigation (clinician-supervised)
Infection/complication monitoring	Q1	Educate patients on warning signs to monitor	False reassurance or unnecessary alarm	Escalation rules + align with clinic aftercare sheet
Hygiene/wound and nasal care	Q2	Reinforce cleaning and care principles	Conflicts with surgeon-specific protocol	"Follow your surgeon's plan" + clinic-approved wording
Bathing/getting nose wet	Q3	Provide timing and precautions guidance	Overgeneralized timelines	Conservative advice + surgeon clearance
Activity/environment restrictions	Q4	Remind avoidance of exertion/irritants	Premature return to activity	Standard restriction ranges + individualized clearance
Symptom relief (swelling/congestion)	Q5	Self-care measures and expectations	Medication nuance missed	Keep medication advice generic; direct to prescriber
Urgent symptoms triage	Q6	Identify red flags requiring contact	Missed emergency symptom	Auto-flag red-flag terms + "contact provider" rule
Healing timeline expectations	Q7	Set realistic recovery expectations	Rigid timelines create false expectations	Present ranges + emphasize variability
Diet/lifestyle support	Q8	General nutrition/hydration advice	Ignores comorbidities	Non-prescriptive; advise clinician input if needed
Sleep positioning	Q9	Head elevation/positioning suggestions	Nonadherence due to discomfort	Practical alternatives + reinforce rationale
Follow-up schedule expectations	Q10	Explain follow-up purpose and visit content	Mismatch with local schedule	State variability; follow surgeon's schedule
